# Actions to Control Hypertension Among Adults in Oklahoma

**Published:** 2010-12-15

**Authors:** Jennifer L. Han

**Affiliations:** Health Care Information, Oklahoma State Department of Health

## Abstract

**Introduction:**

Hypertension is a chronic condition that can be managed with self-monitoring, lifestyle changes, and medication. The purpose of this study was to describe receipt of physician's treatment advice and use of treatments to manage hypertension among Oklahoma's adult population.

**Methods:**

A random-digit–dialed telephone survey was administered to noninstitutionalized adult residents of Oklahoma (n = 7,463) in 2007. Respondents who indicated that they had ever had hypertension (n = 2,937) were asked whether a doctor had advised them on each of 5 general management techniques and whether they used these techniques to manage their condition. Data were weighted and a descriptive analysis of the age-adjusted rates was performed.

**Results:**

Of all hypertensive adults, 91% had received advice from a physician regarding treatment options, and medication was the most commonly recommended therapy (80%). Almost all hypertensive adults were managing their hypertension through use of medication or lifestyle modification, and reducing salt intake was the most common treatment used (74%). Physician advice and individual treatment choices varied by demographic characteristics, although respondents more commonly used a treatment method that was advised by a physician.

**Conclusion:**

Doctors should advise hypertensive patients of treatment options because patients may be more likely to use 1 or more physician-advised options to manage their condition. Efforts should be made to enhance physicians' ability to educate patients about the effects of hypertension and ways in which hypertension can be treated, in addition to enhancing the patients' knowledge of prevention and treatment strategies.

## Introduction

Hypertension is a major risk factor for cardiovascular and kidney disease and a major contributor to premature deaths (1). In 2005-2006, almost 30% of US adults lived with hypertension (systolic blood pressure ≥140 mm Hg or diastolic blood pressure ≥90 mm Hg), and another 28% of US adults had prehypertension (systolic pressure of 120-139 mm Hg or diastolic pressure of 80-89 mm Hg), a condition that puts them at increased risk of developing hypertension (2). Hypertension is a silent disease; as many as 20% of people with hypertension are not aware that they have the condition (2). Irreversible damage can occur in people who are unaware that they have hypertension for years before a diagnosis is made. In people who are aware that they have the disease, complications arise for several reasons: not obtaining physician assistance, not being adequately educated about treatment options (3-5), having uncontrolled blood pressure (1,2,6,7), and failing to adhere to prescribed treatment (8-11).

Prevalence of hypertension and cardiovascular disease, as well as death rates due to cardiovascular and kidney disease, is higher in Oklahoma compared with the rest of the nation (12,13). Furthermore, demographic differences in disease prevalence and mortality exist (2,12,13). Among Oklahoma's adult population, hypertension is more common among those who are older, black, obese, poorer, and less educated (14). Although almost 32% of Oklahoma adults have hypertension (14), how they manage their condition is unknown. Early detection of hypertension and treatment with medication and lifestyle modification may reduce the burden of illness and premature death in Oklahoma (1).

This study was conducted to ascertain how Oklahoma adults who have hypertension control their disease. Hypertensive adults who received treatment advice from a physician were analyzed to determine which treatment methods they used. Treatment options that physicians advised and the actions taken by patients were assessed to determine whether disparities existed among demographic groups.

## Methods

The Behavioral Risk Factor Surveillance System (BRFSS) is the largest ongoing state- and territory-based telephone survey of health behaviors and disease prevalence in the United States. In Oklahoma, BRFSS is coordinated by the Oklahoma State Department of Health, whose in-house call center uses computer-assisted telephone interviewing software to administer the questionnaire to Oklahoma residents aged 18 years or older living in a noninstitutionalized setting. Participants were selected by random-digit–dialing of phone numbers stratified across 6 regions of the state. Only those telephone numbers associated with landlines in residences were considered valid numbers. The BRFSS protocol has exempt status from the institutional review board of the Human Research Protection Office, Department of Health and Human Services under 45 CFR 46.101(b)(2).

Interviewers asked respondents, "Have you ever been told by a doctor, nurse, or other health professional that you have high blood pressure?" If respondents answered yes, interviewers then asked respondents a series of questions regarding how they managed their blood pressure and whether a doctor had advised them of the various treatment options available to manage blood pressure (Appendix). Treatment options included taking medication, modifying diet, reducing salt intake and alcohol consumption, and participating in physical activity.

From January to December 2007, the call center completed interviews with 7,463 noninstitutionalized Oklahoma residents aged 18 years or older. The overall response rate ranged from 49% to 52% per month (15). Data were sent to the Centers for Disease Control and Prevention (CDC) for processing and were returned to the state for analysis. CDC weighted the data, adjusting for noncoverage, nonresponse, and the number of adults and telephones in the household (16).

The researcher analyzed blood pressure management items and physician advice for the 2,937 respondents who indicated that they had hypertension. Mutually exclusive racial and ethnic categories were created (white, black, American Indian not of Hispanic origin, Hispanic, and other); however, in-depth analyses by individual race or ethnicity were not possible, given the small sample sizes in the nonwhite categories. Body mass index was grouped into 3 categories: underweight/normal weight (<25.0 kg/m^2^), overweight (25.0-29.9 kg/m^2^), and obese (≥30.0 kg/m^2^). Respondents who were nonconsumers of salt and alcoholic beverages were excluded from analysis of demographic differences in reducing salt and alcohol consumption, respectively. Records with missing data were excluded from summary analyses specific to the variable to which the missing data pertained.

The researcher age-adjusted the data to the 2000 US standard population (17) and used SAS version 9.2 (SAS Institute, Inc, Cary, North Carolina) and SAS-callable SUDAAN version 10.0 (RTI International, Research Triangle Park, North Carolina) to account for the survey's complex sampling design. Descriptive statistics were determined and pairwise comparisons in age-adjusted rates among groups were evaluated by comparing the 95% confidence intervals. Differences in taking action between respondents who received a physician's advice and those who did not were assessed via *t* tests. Significance was set at α = .05.

## Results

### Characteristics of Oklahoma's hypertensive population

In 2007, 31.5% of Oklahoma adults (n = 2,937) had been told by a health professional that they had hypertension. Most respondents with hypertension were aged 45 years or older and were overweight or obese (Table 1). Almost two-thirds of respondents with hypertension were married, and more than half had lower levels of education (high school diploma/general educational development certification completion or less).

### Doctor's advice to manage blood pressure

Almost 91% of hypertensive adults had received physician counseling regarding treatment options for their condition. Seventeen percent were advised of all treatment options inquired about by BRFSS, and 13.5% were advised of only 1 of the 5 options. Taking medication was the treatment most commonly advised by doctors (Table 2). Almost 10% of hypertensive adults did not receive treatment advice from their doctor.

Differences in age-adjusted rates of treatment advice were evident by sex and age (Table 2). The sole sex-based discrepancy in treatment advice was that doctors more often counseled women than they did men to take medication. Doctors also recommended medication more often as patients' age increased. Doctors more commonly advised patients aged 45 to 64 years to change their diet and exercise, and less frequently advised seniors aged 65 years or older to limit alcohol consumption. There were few socioeconomic differences for type of treatment advice received.

### Individual actions taken to manage blood pressure

Most respondents with hypertension (94.6%) were taking action to manage their condition, and approximately 84% were using more than one type of treatment. Reducing salt intake was the most common treatment being followed (Table 3). Only 5.4% of respondents were not managing their hypertension by using any of the methods inquired about by the BRFSS.

Demographic differences were apparent with respect to methods commonly used to manage blood pressure (Table 3). For example, more women than men took medication as a means of managing blood pressure. Use of medication increased with age, and respondents aged 45 to 64 years were more likely to have modified their diet and reduced alcohol consumption than older adults. A small percentage of obese adults were exercising, and college graduates and respondents with a household income of at least $50,000 were less often reducing alcohol intake than were respondents at lower levels of education and income levels, respectively.

### Following doctor's advice to manage blood pressure

A large proportion of residents who were advised by their doctors to take specific action to manage their blood pressure indicated that they were doing so (Table 4). For instance, 80% of respondents who were advised to take medication used some form of medication, and more than 80% of respondents who were counseled to modify their diet and reduce salt or alcohol intake were following their doctors' advice. Some residents engaged in behaviors to improve their blood pressure without being told to do so by a physician. Reducing salt intake was the most frequent modification made by such people. Of the 10% of people who were not counseled by a physician, approximately one-third were not managing their blood pressure via any method inquired about in the survey.

## Discussion

Of the 31.5% of Oklahoma adults who had hypertension, almost 91% had received advice from a physician regarding how to manage their condition, and approximately 95% were managing their hypertension through using medication, modifying their diet, reducing salt or alcohol intake, or exercising. Medication was the most common treatment advised by a physician, irrespective of demographic, and reducing salt intake was the most common treatment used by the population as a whole. Although demographic differences existed with respect to advice given and treatment used, patterns were not consistent. In general, respondents more often used a specific type of treatment when it was advised by a physician.

The primary reason for managing blood pressure is to reduce illness and death related to heart disease, stroke, and kidney disease (1,2). Medication is the primary treatment for hypertension, and several classes of medications can be taken to assist with lowering blood pressure. Most people require more than 1 medication to control their condition (1) and may use different medications before finding the most effective one. Because medications are key to reducing hypertension and its associated risk of stroke and other debilitating events and taking medication may be simpler to accomplish than incorporating several lifestyle changes (11), it was not surprising that medication was the most commonly advised treatment among Oklahomans. It was also the most commonly used treatment for adults aged 45 years or older, perhaps because of its effectiveness at reducing risk of chronic diseases associated with hypertension and because older adults may have difficulty managing their condition (18,19). However, the BRFSS survey did not include questions about type and number of medications being used, adherence to prescribed treatment, and whether blood pressure was under control.

Lifestyle modifications are necessary to prevent and manage hypertension. Such modifications include dietary changes (ie, adopting the DASH [Dietary Approaches to Stop Hypertension] eating plan, which involves eating more fruits, vegetables, and low-fat dairy products and fewer saturated fats), reducing sodium and alcohol consumption, engaging in regular physical activity, and maintaining a healthy weight (1). Of the lifestyle treatments inquired about by BRFSS, exercising and reducing salt intake were most commonly advised for Oklahoma adults, followed by making dietary modifications. Although these lifestyle treatments enable modest reductions in blood pressure, weight loss and weight control can have a greater effect on blood pressure (1) and may lower the risk of developing hypertension for people who do not already have the condition (1,20,21). Studies have demonstrated that weight loss and weight control are prominent treatments advised by physicians to their patients (3,4), yet questions regarding weight control as a treatment for hypertension were not included in the BRFSS survey. Modifying the diet and engaging in physical activity are actions that may lead to weight loss, however, and should be recommended to people who are overweight and obese. Both of these lifestyle treatments were advised more commonly for hypertensive Oklahomans who were obese, a finding similar to that of Mellen et al (5), although results from the Oklahoma BRFSS were not significant.

In general, people more often used a treatment that was recommended by a physician. This observation underscores the importance of supplying people who have been diagnosed with hypertension with enough information to make informed decisions, including strategies to assist them in making key lifestyle changes. Overall, 10% of hypertensive Oklahomans received no physician advice, and almost one-third of these people were not managing their blood pressure via using medication, making dietary changes, reducing salt and alcohol intake, or exercising, which puts them at higher risk of developing cardiovascular and kidney diseases (2). Of respondents who received advice from a physician, few were informed of all available treatment options inquired about in the BRFSS survey, although advice rates for lifestyle modifications were much higher than those observed in other studies (3,5).

There are several possible reasons why patients were not advised of all treatment options, including having a less severe condition or lack of other risk factors. Perhaps some physicians did not have sufficient time to spend with patients (22), underestimated their risk (23), or believed that patients do not listen or understand the problem (22). Physicians may be less likely to provide lifestyle recommendations and intensive counseling because they lack the training in lifestyle counseling to do so (3,5). Also, patients may not remember having received information regarding a specific treatment and thus would not have responded positively to the BRFSS survey questions regarding treatment advice. Regardless, not fully educating patients on all available treatments may affect their ability to make well-informed decisions and inhibits the ability to experiment with various treatments to find the single treatment or combination of treatments that is most successful. Even considering differences in patients' health histories and disease severity, lifestyle modifications alone would benefit patients' overall health, particularly their cardiovascular health.

Managing hypertension is difficult, and as many as 65% of people in the United States do not have good control over their condition, meaning their blood pressure is not maintained below 140/90 mm Hg (7). Physicians should educate patients on treatment options and the consequences of leaving hypertension untreated, and patients should adhere to their doctors' advice. Adherence, sometimes referred to as compliance, is estimated to be 50% for medication use and even lower for behavior modifications (11). Although a large percentage of respondents were estimated to have followed their physicians' treatment advice, adherence rates could not be determined with the BRFSS survey. Fewer people were likely actually adhering to their physicians' recommendations than was estimated.

Recommendations for improving patient adherence to treatment have been published (1). Advice from a physician may act as a primer for forthcoming information, improve recall, improve the sharing of information with others, and perhaps effect behavior change (24). Physicians should discuss the consequences of allowing hypertension to go untreated, as they sometimes do not (3). Advice is not enough to effect compliance and long-term adherence, however. The consensus is that patients must be motivated to adhere to a regimen, and although patients should take personal responsibility for their actions, they also require education, reinforcement, individualized programs, monitoring, and other types of assistance from health care professionals to ensure successful treatment (1,18,20,25). Health promotion efforts that target physicians in an effort to improve their rates of providing advice, introduce novel ways of educating their patients, and assist them in increasing adherence would benefit both the physician and patient.

There are several strengths to this study. The sample was a stratified random sample of Oklahoma's noninstitutionalized adult population. Data were weighted to reduce bias and to provide a more accurate representation of the population from which the sample was drawn. Statistical analysis used methods most appropriate for weighted data.

There are also some limitations to this study. Households without landline telephones were not included in the 2007 survey, and people who live in cell-phone-only households may have different health risks and behaviors than people who live in households with landline service. Respondents may have provided answers to questions that they thought would be more appropriate, potentially introducing social desirability bias to the data, which tends to overreport good behavior and underreport bad behavior. Because BRFSS surveys a cross-section of the population, associations rather than cause-and-effect relationships were assessed. Comorbidities were not evaluated, and the survey did not include questions about when the respondents' last blood pressure screening occurred, whether they currently had high blood pressure, whether they were truly compliant with their physicians' advice, and whether their blood pressure was under control.

In summary, almost 91% of Oklahoma adults with hypertension had received advice from a physician regarding how to manage their condition, and approximately 95% were managing their hypertension through some combination of using medication, modifying diet, reducing salt or alcohol intake, or exercising. Respondents who received advice from a doctor about a specific type of treatment had higher rates of using that type of treatment. Therefore, efforts should be made to enhance physicians' ability to educate patients about the effects of hypertension and ways in which hypertension can be treated.

## Figures and Tables

**Table 1:** Demographic Characteristics of Survey Respondents Who Have Hypertension (N = 2,937), Behavioral Risk Factor Surveillance System, Oklahoma, 2007

**Characteristics**	Sample Size^a^(N = 2,937)	Weighted %^b^(95% CI)
**Sex**		
Male	1,035	50.2 (47.9-52.5)
Female	1,902	49.8 (47.5-52.1)
**Age, y**		
18-44	320	21.2 (18.8-23.6)
45-64	1,174	43.7 (41.4-46.0)
≥65	1,443	35.1 (33.2-37.1)
**Body mass index, kg/m^2^**		
<25.0	701	21.1 (19.3-23.0)
25.0-29.9	1,034	37.3 (35.0-39.6)
≥30.0	1,075	41.6 (39.2-43.9)
**Race/ethnicity**		
White	2,253	70.7 (68.5-73.0)
Black	202	8.3 (6.9-9.6)
American Indian	186	9.3 (7.6-10.9)
Hispanic	80	4.3 (3.2-5.4)
Other	208	7.4 (6.2-8.6)
**Marital status**		
Married	1,572	65.7 (63.5-67.8)
Unmarried	1,359	34.3 (32.2-36.5)
**Education**		
High school diploma or less	1,518	51.1 (48.8-53.4)
Some college/technical school	817	28.1 (26.0-30.1)
College graduate	597	20.8 (19.0-22.6)
**Household income, $**		
<25,000	1,097	38.2 (35.8-40.6)
25,000-49,999	734	29.3 (27.1-31.5)
≥50,000	680	32.5 (30.1-34.8)

Abbreviation: CI, confidence interval.

a Some cells may not add to 2,937 because of missing data. Records with missing data were excluded from summary analyses specific to the variable to which the missing data pertained.

b Data were weighted and adjusted for noncoverage, nonresponse, and the number of adults and telephones in the household.

**Table 2:** Frequencies of Survey Respondents and Age-Adjusted Estimates of Hypertensive Adults Who Were Advised by a Doctor of Treatments, Behavioral Risk Factor Surveillance System, Oklahoma, 2007

Characteristic	Treatment Advised, Weighted %^a^ (95% Confidence Interval)				

	Take Medication	Modify Diet	Reduce Salt Intake^b^	Reduce Alcohol Intake^c^	Exercise
Total (n = 2,937)	80.1 (75.9-83.7)	59.8 (55.7-63.7)	69.4 (65.5-73.0)	33.0 (29.2-37.0)	68.2 (64.1-72.0)
**Sex**					
Male (n = 1,035)	75.9 (69.8-81.1)	60.0 (54.1-65.6)	68.4 (62.7-73.7)	37.4 (31.9-43.2)	67.0 (61.0-72.4)
Female (n = 1,902)	86.6 (82.1-90.1)	61.3 (56.6-65.9)	70.8 (66.1-75.0)	27.8 (23.4-32.8)	71.3 (66.8-75.5)
**Age, y**					
18-44 (n = 320)	66.7 (59.1-73.6)	58.6 (51.1-65.7)	67.2 (60.1-73.6)	39.7 (32.9-47.0)	64.3 (56.8-71.2)
45-64 (n = 1,174)	94.0 (92.2-95.4)	67.5 (64.3-70.6)	73.2 (70.1-76.1)	31.0 (27.8-34.3)	75.7 (72.8-78.5)
≥65 (n = 1,443)	97.5 (96.4-98.3)	49.7 (46.7-52.8)	69.4 (66.5-72.1)	15.5 (13.3-17.9)	66.9 (64.1-69.6)
**BMI, kg/m^2^**					
<25.0 (n = 701)	—d	—	—	—	—
25.0-29.9 (n = 1,034)	79.1 (71.9-84.9)	55.8 (49.0-62.4)	69.9 (63.4-75.6)	30.1 (24.1-37.0)	64.7 (57.7-71.2)
≥30.0 (n = 1,075)	82.3 (75.9-87.3)	67.2 (60.9-72.9)	70.0 (64.2-75.3)	34.7 (29.0-40.9)	74.3 (68.4-79.5)
**Education**					
High school diploma or less (n = 1,518)	78.7 (72.4-83.9)	56.3 (50.3-62.1)	69.6 (63.6-74.9)	34.2 (28.6-40.2)	64.0 (58.0-69.5)
Some college/technical school (n = 817)	83.5 (76.3-88.8)	67.6 (60.8-73.7)	68.2 (61.2-74.4)	35.7 (29.0-43.0)	75.3 (68.7-81.0)
College graduate (n = 597)	79.6 (70.2-86.6)	59.0 (50.6-66.9)	70.2 (63.3-76.3)	26.2 (20.2-33.2)	70.1 (61.0-77.9)
**Annual income, $**					
<25,000 (n = 1,097)	79.9 (72.4-85.7)	59.9 (53.0-66.5)	74.5 (68.3-79.9)	35.8 (29.4-42.8)	68.5 (62.0-74.3)
25,000-49,999 (n = 734)	82.0 (73.3-83.4)	60.4 (52.0-68.2)	70.5 (62.4-77.5)	39.4 (31.6-47.7)	68.8 (60.1-76.4)
≥50,000 (n = 680)	81.6 (74.4-87.2)	62.4 (55.5-68.9)	67.6 (61.2-73.4)	28.9 (23.6-34.9)	74.7 (67.6-80.6)

Abbreviation: BMI, body mass index.

a Data were weighted and adjusted for noncoverage, nonresponse, and the number of adults and telephones in the household.

b Weighted percentages include only participants who used salt (n = 2,843).

c Weighted percentages include only participants who consumed alcohol (n = 1,634).

d Records with missing data were excluded from summary analyses specific to the variable to which the missing data pertained. A dash (—) indicates an unstable rate, with standard error >5.0.

**Table 3:** Frequencies of Survey Respondents and Age-Adjusted Estimates of Hypertensive Adults Who Took Action to Manage Their Blood Pressure, Behavioral Risk Factor Surveillance System, Oklahoma, 2007

Characteristic	Treatment Advised, Weighted %^a^ (95% Confidence Interval)				

	Took Medication	Modified Diet	Reduced Salt Intake^b^	Reduced Alcohol Intake^c^	Exercised
Total (n = 2,937)	66.1 (62.3-69.6)	66.4 (62.4-70.2)	74.2 (70.2-77.8)	59.8 (54.6-64.8)	63.5 (59.8-67.1)
**Sex**					
Male (n = 1,035)	61.9 (56.9-66.7)	65.8 (60.0-71.2)	73.0 (67.4-78.3)	59.6 (52.5-66.2)	66.1 (60.6-71.2)
Female (n = 1,902)	73.0 (68.1-77.4)	67.1 (62.3-71.6)	75.6 (70.9-79.7)	61.2 (54.6-67.4)	60.5 (55.7-65.1)
**Age, y**					
18-44 (n = 320)	46.3 (39.7-53.1)	63.8 (56.5-70.5)	70.7 (63.4-77.2)	58.0 (48.8-66.8)	67.0 (60.1-73.3)
45-64 (n = 1,174)	84.2 (81.6-86.4)	73.5 (70.4-76.3)	78.5 (75.4-81.2)	68.6 (63.8-73.0)	60.5 (57.1-63.8)
≥65 (n = 1,443)	95.8 (94.4-96.8)	62.2 (59.2-65.1)	77.3 (74.5-79.8)	49.8 (44.2-55.4)	58.1 (55.1-61.0)
**BMI, kg/m^2^**					
<25.0 (n = 701)	59.8 (51.1-67.9)	—d	—	—	—
25.0-29.9 (n = 1,034)	63.0 (56.8-68.7)	68.4 (61.9-74.2)	74.1 (67.5-79.8)	53.0 (44.5-61.3)	71.6 (66.1-76.6)
≥30.0 (n = 1,075)	70.2 (64.3-75.5)	66.1 (60.1-71.6)	75.2 (69.1-80.4)	66.4 (58.9-73.2)	56.6 (50.8-62.3)
**Education**					
High school diploma or less (n = 1,518)	62.3 (57.0-67.3)	63.2 (57.3-68.7)	74.8 (68.6-80.2)	64.6 (57.0-71.6)	59.9 (54.2-65.3)
Some college/technical school (n = 817)	68.1 (61.2-74.4)	69.3 (62.3-75.4)	72.6 (65.3-78.9)	66.4 (57.7-74.1)	65.8 (59.2-71.9)
College graduate (n = 597)	73.2 (64.8-80.2)	70.8 (62.7-77.8)	74.5 (67.7-80.2)	43.6 (34.4-53.3)	69.4 (62.4-75.6)
**Annual income, $**					
<25,000 (n = 1,097)	61.9 (55.7-67.7)	66.6 (59.9-72.7)	80.7 (74.2-85.9)	72.8 (64.1-80.1)	61.4 (55.2-67.3)
25,000-49,999 (n = 734)	70.7 (62.5-77.7)	68.8 (60.5-76.0)	74.9 (66.5-81.8)	66.2 (56.7-74.5)	61.8 (53.4-69.6)
≥50,000 (n = 680)	69.3 (62.7-75.2)	67.7 (61.1-73.7)	70.9 (64.4-76.6)	47.6 (39.7-55.6)	69.5 (63.5-74.9)

Abbreviation: BMI, body mass index.

a Data were weighted and adjusted for noncoverage, nonresponse, and the number of adults and telephones in the household.

b Weighted percentages include only those who used salt (n = 2,685).

c Weighted percentages include only those who consumed alcohol (n = 1,230).

d Records with missing data were excluded from summary analyses specific to the variable to which the missing data pertained. A dash (—) indicates an unstable rate, with standard error > 5.0.

**Table 4:** Frequencies of Survey Respondents and Age-Adjusted Estimates of Oklahoma's Hypertensive Residents Who Followed a Doctor's Advice or Managed Blood Pressure On Their Own, Behavioral Risk Factor Surveillance System, Oklahoma, 2007^a^

Treatment	Advised by Doctor		Not Advised by Doctor		*P* Value

	Sample Size^b^n/N	Weighted %^c^(95% CI)	Sample Size^b^n/N	Weighted %^c^(95% CI)	
Took medication	2,399/2,614	80.3 (76.0-83.9)	25/174	11.8 (8.1-16.9)	<.05
Modified diet	1,238/1,544	80.4 (76.3-84.0)	584/1,211	46.2 (39.4-53.1)	<.05
Reduced salt intake^d^	1,396/1,602	84.8 (79.9-88.6)	515/886	57.1 (50.3-63.6)	<.05
Reduced alcohol intake^e^	273/324	81.3 (73.1-87.4)	307/674	47.8 (41.2-54.6)	<.05
Exercised	1,256/1,880	71.3 (67.3-75.0)	373/875	47.6 (40.3-55.0)	<.05

Abbreviation: CI, confidence interval.

a Records with missing data were excluded from summary analyses specific to the variable to which the missing data pertained.

b Sample size refers to the number of respondents who engaged in the action (n)/number of respondents who were advised of the treatment method (N).

c Data were weighted and adjusted for noncoverage, nonresponse, and the number of adults and telephones in the household.

d Weighted percentages include only those who used salt (n = 2,685).

e Weighted percentages include only those who consumed alcohol (n = 1,230).

## Appendix. Questions From the Core Survey and Optional Module, Behavioral Risk Factor Surveillance System, Oklahoma, 2007

Respondents who answered yes when asked, "Have you ever been told by a doctor, nurse, or other health professional that you have high blood pressure?," were asked the questions listed below. Possible responses for each item were yes, no, "don't know/not sure," and "refused." The items pertaining to salt and alcohol also included the responses "do not use salt" and "do not drink," respectively.

**From the Core Survey**

Question: Are you currently taking medicine for your high blood pressure?

**From the Optional Module**

**Preface:** Are you now doing any of the following to help lower or control your high blood pressure?

**Question:** [Are you] changing your eating habits (to help lower or control your high blood pressure)?

**Question:** [Are you] cutting down on salt (to help lower or control your high blood pressure)?

**Question:** [Are you] reducing alcohol use (to help lower or control your high blood pressure)?

**Question:** [Are you] exercising (to help lower or control your high blood pressure)?

**Preface:** Has a doctor or other health professional ever advised you to do any of the following to help lower or control your high blood pressure?

**Question:** [Ever advised you to] change your eating habits (to help lower or control your high blood pressure)?

**Question:** [Ever advised you to] cut down on salt (to help lower or control your high blood pressure)?

**Question:** [Ever advised you to] reduce alcohol use (to help lower or control your high blood pressure)?

**Question:** [Ever advised you to] exercise (to help lower or control your high blood pressure)?

**Question:** [Ever advised you to] take medication (to help lower or control your high blood pressure)?
